# Miscellaneous Intelligence

**Published:** 1846-04-01

**Authors:** 


*Miscellaneous Intelligence.*—The Medical class at Willoughby medical college, passed some highly complimentary resolutions at the close of the lectures on Materia Medica, by Prof. T. Rush Spencer.—The Pope of Rome has forbidden his subjects from attending any scientific congress, and physicians are not allowed to continue their attendance on patients who do not receive the sacrament after the third visit.—A new medical college has been organized in Philadelphia, entitled the "Franklin Medical College." The announcement for the session of 1846-7 states that it has been established "under the opinion that increased accomodation was demanded for the great concourse of students now resorting to Philadelphia" (!) The teachers are Anatomy and Histology, Paul B. Goddard, M. D.; Surgery, C. C. Van Wyck, M. D.; Medicine, Meredith Clymer, M. D.; Materia Medica, John B. Biddle, M. D.; Obstetrics, David H. Tucker, M. D.; Physiology and Legal Medicines, Leven S. Jones, M. D.; general organic Chemistry, James B. Rogers, M. D. Philadelphia has now four medical schools. — We find the following very candid confession in one of our city newspapers: "water cure — caution. — The water cure Journal of Dr. Shew, of New-York, in an article on using water in consumption, gives this caution: — "It is to be remembered that we cannot harden every person, and that we may kill many in the attempt."—Prof. Dudley of Lexington, Ky., the most eminent and successful Lithotomist living, has performed the operation in 185 cases—180 of which terminated favorably. Dr. Bush in a late article in the Western Lancet, denies that these were *selected* cases.—A friend has directed our attention to a communication in the New-York Herald, giving some account of the address delivered at the med. dept., of the University, on the occasion of conferring medical degrees. The writer states that the professor occupied a good portion of the hour in remarks upon the approaching national convention, which he ridiculed as the project of a young man from Binghamton! We hope the address will be published. We should like to see what the professor has to say respecting the atrocious crime of being a young man, and the additional enormity of being a young man from Binghamton!—A new edifice for the medical college in Boston is about to be erected, the site having been presented by Dr. Parkman of that city. Increased accomodations have long been needed.

